# A cautionary note on the naive use of general-population biobanks to study pulmonary arterial hypertension, with a focus on Mendelian randomisation

**DOI:** 10.1183/13993003.00436-2025

**Published:** 2025-10-16

**Authors:** Benjamin Woolf, Eckart De Bie, Vallerie McLaughlin, Stefan Gräf, Mark Toshner, Martin R. Wilkins, Christopher J. Rhodes, Stephen Burgess

**Affiliations:** 1 The MRC Integrative Epidemiology Unit, University of Bristol, Bristol, UK; 2 School of Psychological Science, University of Bristol, Bristol, UK; 3 The MRC Biostatistics Unit, University of Cambridge, Cambridge, UK; 4 Victor Phillip Dahdaleh Heart and Lung Research Institute, Department of Medicine, University of Cambridge, Cambridge, UK; 5 Royal Papworth Hospital NHS Foundation Trust, Cambridge, UK; 6 Division of Cardiovascular Medicine, Department of Internal Medicine, University of Michigan, Ann Arbor, MI, USA; 7 National Heart and Lung Institute, Imperial College London, London, UK; 8 British Heart Foundation Cardiovascular Epidemiology Unit, Department of Public Health and Primary Care, University of Cambridge, Cambridge, UK

## Abstract

Pulmonary hypertension (PH) is defined by a mean pulmonary artery pressure >20 mmHg [1]. Patients with PH are assigned to one of five internationally recognised groups. Pulmonary arterial hypertension (PAH), or group 1 PH, is a heterogeneous collection of conditions characterised by increased precapillary pulmonary vascular resistance. Groups 2 to 5 PH comprise PH caused, in turn, by left heart disease, lung diseases (*e.g.* COPD), chronic thromboembolism, and miscellaneous causes such as haematological diseases.

## Shareable abstract

**There are two issues with using existing data from general-population biobanks to study PAH: low power and non-random misclassification. Relative to gold standard data, this results in false-positive and false-negative findings.** https://bit.ly/4nksm6c

*To the Editor:*

Pulmonary hypertension (PH) is defined by a mean pulmonary artery pressure >20 mmHg [1]. Patients with PH are assigned to one of five internationally recognised groups. Pulmonary arterial hypertension (PAH), or group 1 PH, is a heterogeneous collection of conditions characterised by increased precapillary pulmonary vascular resistance. Groups 2 to 5 PH comprise PH caused, in turn, by left heart disease, lung diseases (*e.g.* COPD), chronic thromboembolism, and miscellaneous causes such as haematological diseases.

PAH is increasingly being investigated using data from biobanks with a general population sampling frame (figure 1a), as opposed to disease-specific cohorts. General population biobanks tend to define PAH with a single medical record code. Many studies use these data to perform Mendelian randomisation (MR; figure 1b). MR is a study design that uses genetic variants specifically associated with an exposure of interest to test causal claims [2, 3]. We demonstrate two issues with existing general population biobank PAH data: low power and non-random misclassification. These result in a failure to replicate findings from gold standard PAH datasets in general population biobanks, and spurious findings from general population biobanks that fail to replicate in gold standard datasets.

**FIGURE 1:**
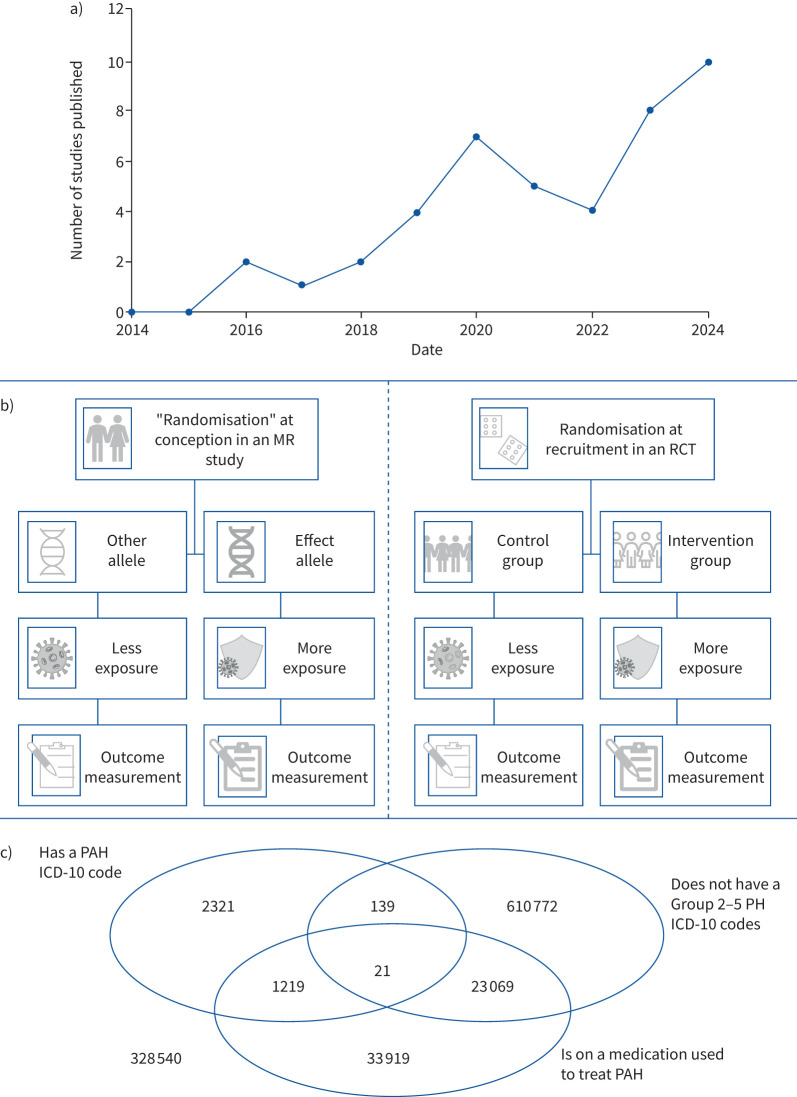
a) Results of a search for: “pulmonary arterial hypertension” AND (“biobank” OR “UK Biobank” OR “UKB” OR “UKBB” OR “FinnGen”) between 1 January 2014 and 31 December 2024 in PubMed. b) Illustration of the analogy leveraged in Mendelian randomisation (MR) investigations between genetic inheritance and randomisation in a randomised controlled trial (RCT): in an RCT, participants are randomised to receive an intervention which changes the exposure or a placebo and then followed up over time. Analogously, in a MR investigation, participants randomly inherit at conception an effect allele which impacts the exposure of interest, or a non-effect allele which does not. We would encourage readers interested in learning more about MR to read the primers cited [2, 3]. c) Venn diagram with the frequency per million of the pulmonary arterial hypertension (PAH) ICD-10 code I27.0, prescriptions of medication used to treat PAH (see below; sildenafil, tadalafil, selexipag, ambrisentan, bosentan, macitentan, epoprostenol, iloprost, treprostinil and sotatercept), and not having ICD-10 codes related to groups 2 to 5 pulmonary hypertension (PH; C96, D18.1, D57, D69.0, D73, D86, E01, E02, E03, E04, E05, E06, E07, E74.0, E75.2, G47.3, I11, I26, I27.8, I34, I35, I50, J40, J41, J42, J43, J80, J81, J82, J84, J98.5, L95, L94, Q85.0, R06.8, T70,W94 and Y84.1) among individuals with electronic health record data in the All of Us biobank (version 7). The category “on a medication to treat PAH” includes individuals taking at least one of the listed drugs. While calcium channel blockers are used to treat some PAH patients, they are rarely used as a monotherapy treatment and are highly non-specific to PAH, so were not included in this list. Some of the other listed medications are also used for other conditions (for example, sildenafil for erectile dysfunction), and so many of the individuals listed as “on a medication to treat PAH” may not have PAH.

Because PAH is rare, with a prevalence below 50 cases per million [1], population-based biobanks have few cases. Power in case–control studies is not substantially improved by increasing the case-to-control ratio beyond 1:4 [4]. Consequently, biobanks are typically less well powered than PAH-specific cohorts, despite having tens or hundreds of times more participants.

Low power has two implications: true-positive associations are more likely to be missed, and detected associations have lower odds of being true. Indeed, a reported association between variants proxying *IL6R* signalling and PAH risk observed in an early release of FinnGen failed to replicate in much larger cohorts [5].

Meta-analyses can address low power. Researchers therefore meta-analysed three general-population biobanks (UK Biobank (UKB), FinnGen release 12, and Million Veteran Program (MVP)) with 3302 apparent PAH cases and 1 205 457 controls (https://mvp-ukbb.finngen.fi/pheno/I9_HYPTENSPUL) [6–8]. The prevalence in this meta-analysis is much higher than expected based on general population surveys. This might imply the presence of misclassification.

Misclassification (when people categorised as cases do not have the condition, or people categorised as controls do) reduces power [9]. The Rhodes *et al.* [10] genome-wide association study (GWAS) contained 2085 PAH cases with gold standard expert centre diagnoses, and 9659 controls. The FinnGen-UKB-MVP meta-analysis is theoretically better powered because it has more “cases” and controls. Failure to detect an association observed in Rhodes *et al.* [10] in the FinnGen-UKB-MVP meta-analysis would suggest misclassification of cases in the general population biobanks.

Rhodes *et al.* [10] detected associations with three independent variants at genome-wide significance (p<5×10^−8^): rs2856830 in the *HLA-DPA1/DPB1* gene cluster, and rs13266183 and rs10103692 near the *SOX17* gene. The *SOX17* gene was then followed up in cell and animal models, which demonstrated the functional relevance of the variants. However, none of these variants are associated with PAH in the FinnGen-UKB-MVP meta-analysis (p=0.506 for rs2856830, p=0.241 for rs13266183, and p=0.318 for rs10103692). This strongly suggests that the general population biobanks do not accurately identify PAH cases.

Although not ideal, when misclassification is random, information can still be gained from large numbers of noisy observations [9]. However, non-random misclassification can introduce bias. Rare conditions like PAH are susceptible to misclassification bias in general population biobanks because a tiny percentage of controls that are non-randomly misclassified can overwhelm signal from the limited number of true cases. (Correctly classifying 99.99% of non-PAH individuals in a general population sample will result in 100 false cases per million, twice the prevalence of true cases.)

One possible source of non-random misclassification is individuals with non-group 1 PH who incorrectly receive a PAH medical record code. We examined this by testing if causes of groups 2 to 5 PH associate with PAH diagnosis in MR analyses. We used GWASs on pulmonary emboli (32 876 cases, 1 508 902 controls) and left heart failure (10 857 cases, 1 463 784 controls) from the FinnGen-UKB-MVP meta-analysis, and COPD (58 559 cases, 937 358 controls) from the Global Biobank Meta-Analysis [11]. Cases were defined using medical record codes. We selected genetic proxies for each phenotype using independent (clumping r^2^=0.001 and distance=10 Mb) genome-wide significant variants, and meta-analysed MR Wald ratios using an inverse variance weighting. The MR analysis using the FinnGen-UKB-MVP meta-analysis found evidence supporting genetically predicted left heart failure (OR 1.916, 95% CI 1.660 to 2.210), COPD (OR 1.376, 95% CI 1.092 to 1.734) and pulmonary emboli (OR 1.110, 95% CI 1.016 to 1.212) as risk factors for PAH diagnoses. This was not replicated by Rhodes *et al.* [10] (OR 1.209, 95% CI 0.967 to 1.512; OR 1.144, 95% CI 0.772 to 1.697; and OR 0.997, 95% CI 0.874 to 1.137, respectively).

These differences can be explained by case misclassification. Alternative explanations are not convincing. Failure to replicate may be due to case misclassification. An alternative explanation is lower power; however, this would only lead to wider confidence intervals, whereas we also see substantial attenuation in estimates. While there are demographic differences between Rhodes *et al.* [10] and the FinnGen-UKB-MVP meta-analysis, the differences between the biobanks are at least as large. Thus, if demographic differences are important, there should also be heterogeneity in estimates between the biobanks. However, substantial heterogeneity in PAH associations was not observed (minimum heterogeneity p_FDR_=0.089).

Non-random misclassification can bias downstream analyses. To illustrate, atrial fibrillation (AF) is associated with left heart failure but does not cause PAH. Misclassification of group 2 PH might create a false-positive AF–PAH association. Selecting variants (using the same parameters described above) from the FinnGen-UKB-MVP meta-analysis of AF (170 643 cases and 1 163 021 controls), we observe an association of genetically predicted AF with PAH in the FinnGen-UKB-MVP PAH meta-analysis (p<0.001) but not in Rhodes *et al.* [10] (p=0.457).

Non-PH individuals can also be misclassified with PAH, for example due to a related cardiorespiratory disease. In the All of Us biobank there are 21 individuals per million who had a PAH ICD-10 code, were on a medication used to treat PAH, and had no group 2 to 5 related ICD-10 codes [12]. However, 129 individuals per million have a PAH ICD-10 code but are not on any PAH medication and do not have a group 2 to 5 PH medical record code (figure 1c); these likely largely represent non-PH individuals with an incorrect PAH diagnosis.

Researchers wishing to use general population biobanks to study rare diseases such as PAH need to ensure they carefully address misclassification. Because PAH cases are unlikely to be unmedicated, the literature suggests supplementing medical records with PAH-related prescriptions and/or requiring elevated pulmonary pressures [13]. However, since non-group 1 PH individuals are prescribed PAH medication, requiring cases to use PAH medication may not address misclassification between PH groups. The blanket exclusion of people with conditions related to group 2 to 5 PH will also exclude true PAH cases due to high co-occurrence of some of these conditions in gold standard-diagnosed PAH patients [14].

To advance the field, a call-to-action was initiated by the Genetics and Genomics Task Force 3 at the 7th World Symposium on Pulmonary Hypertension to assemble a global, diverse, condition-specific genetic registry [15]. We would encourage researchers or clinicians with genotyped PH patients to support the registry.

To conclude, general population biobanks defining PAH with a single medical record code are likely to produce unreliable results due to non-random misclassification and low power. When misclassification is truly random, it can be addressed by increasing sample sizes. Non-random misclassification cannot be addressed so simply. The spurious MR findings presented here highlight that even “robust” designs require good quality data. Inaccurate PAH definitions are thus likely to bias conventional observational analyses. Currently available general population biobank GWASs of PAH should therefore be avoided in downstream analyses such as MR.

## Shareable PDF

10.1183/13993003.00436-2025.Shareable1 This PDF extract can be shared freely online.
Shareable PDF

ERJ-00436-2025.Shareable

## Data Availability

GWAS data from the publication by C.J. Rhodes and co-workers are available from https://www.ebi.ac.uk/gwas/publications/30527956, Global Biobank Meta-analysis summary statistics are available from https://www.globalbiobankmeta.org/, and FinnGen meta-analysis summary statistics are available from https://www.finngen.fi/en/access_results. The R code used in this study is available from https://github.com/bar-woolf/applied-MR-code/blob/main/PAH%20biobank%20research%20letter%20code%20shareable.R.
