# Supplementary material for: A cautionary note on the naive use of general-population biobanks to study pulmonary arterial hypertension, with a focus on Mendelian randomisation
Source: Eur Respir J. 2025 Oct 16;66(4):2500436. doi: 10.1183/13993003.00436-2025 (PMC12528775; doi:10.1183/13993003.00436-2025)
Supplement: Supplementary file 1 [file ERJ-00436-2025.Shareable.pdf]

EUROPEAN RESPIRATORY JOURNAL
RESEARCH LETTER
B. WOOLF ET AL.

A cautionary note on the naive use of general-population biobanks to study pulmonary arterial hypertension, with a focus on Mendelian randomisation

Benjamin Woolf [1,2,3], Eckart De Bie [4,5], Vallerie McLaughlin[6], Stefan Gräf [4], Mark Toshner [4,5], Martin R. Wilkins [7], Christopher J. Rhodes[7] and Stephen Burgess[3,8]

[1]The MRC Integrative Epidemiology Unit, University of Bristol, Bristol, UK. [2]School of Psychological Science, University of Bristol, Bristol, UK. [3]The MRC Biostatistics Unit, University of Cambridge, Cambridge, UK. [4]Victor Phillip Dahdaleh Heart and Lung Research Institute, Department of Medicine, University of Cambridge, Cambridge, UK. [5]Royal Papworth Hospital NHS Foundation Trust, Cambridge, UK. [6]Division of Cardiovascular Medicine, Department of Internal Medicine, University of Michigan, Ann Arbor, MI, USA. [7]National Heart and Lung Institute, Imperial College London, London, UK. [8]British Heart Foundation Cardiovascular Epidemiology Unit, Department of Public Health and Primary Care, University of Cambridge, Cambridge, UK.

Corresponding author: Benjamin Woolf (benjamin.woolf@bristol.ac.uk)

Shareable abstract (@ERSpublications)
There are two issues with using existing data from general-population biobanks to study PAH: low power and non-random misclassification. Relative to gold standard data, this results in false-positive and false-negative findings. https://bit.ly/4nksm6c

Cite this article as: Woolf B, De Bie E, McLaughlin V, *et al.* A cautionary note on the naive use of general-population biobanks to study pulmonary arterial hypertension, with a focus on Mendelian randomisation. *Eur Respir J* 2025; 66: 2500436 [DOI: 10.1183/13993003.00436-2025].

This PDF extract can be shared freely online.

Copyright ©The authors 2025

This version is distributed under the terms of the Creative Commons Attribution Licence 4.0.

Received: 6 March 2025
Accepted: 29 Aug 2025

To the Editor:

Pulmonary hypertension (PH) is defined by a mean pulmonary artery pressure >20 mmHg [1]. Patients with PH are assigned to one of five internationally recognised groups. Pulmonary arterial hypertension (PAH), or group 1 PH, is a heterogeneous collection of conditions characterised by increased precapillary pulmonary vascular resistance. Groups 2 to 5 PH comprise PH caused, in turn, by left heart disease, lung diseases (*e.g.* COPD), chronic thromboembolism, and miscellaneous causes such as haematological diseases.
